# Development, Validation, and Reproducibility of Food Group-Based Frequency Questionnaires for Clinical Use in Brazil: A Pre-Hypertension and Hypertension Diet Assessment

**DOI:** 10.3390/nu13113881

**Published:** 2021-10-29

**Authors:** Sinara L. Rossato, Francisca Mosele, Leila B. Moreira, Marcela Perdomo Rodrigues, Ruchelli França Lima, Flávio D. Fuchs, Sandra C. Fuchs

**Affiliations:** 1 Postgraduate Program of Epidemiology, School of Medicine, Universidade Federal do Rio Grande do Sul, Porto Alegre 90035-003, RS, Brazil; franciscamosele@nutritionthinking.com (F.M.); lb_moreira@yahoo.com.br (L.B.M.); ffuchs@hcpa.edu.br (F.D.F.); sfuchs@hcpa.edu.br (S.C.F.); 2 Institute of Geography, Campus Santa Mônica, Universidade Federal de Uberlândia, Uberlândia 38408-100, MG, Brazil; 3 Department of Nutrition, Harvard T.H. Chann School of Public Health, Harvard University, Boston, MA 02115, USA; 4 PREVER National Institute of Science and Technology, Hospital de Clínicas de Porto Alegre, Porto Alegre 90035-903, RS, Brazil; ruchelliflima@gmail.com; 5 Nutrition Thinking® Co., Tecnopuc, Pontifícia Universidade Católica do Rio Grande do Sul, Porto Alegre 90619-900, RS, Brazil; 6 Postgraduate Studies Program in Cardiology, School of Medicine, Universidade Federal do Rio Grande do Sul, Porto Alegre 91509-900, RS, Brazil; perdomomr@gmail.com

**Keywords:** food intake, diet, diet assessment, diet intake, food frequency questionnaire, outpatient clinic, pre-hypertension, hypertension, PREVER study

## Abstract

The Blood pressure control diet is well described; however, it has not been implemented in clinical care, possibly due to the impracticability of the diet assessment in these contexts. In order to facilitate the dietary assessment, we developed and assessed the validity and reproducibility of two food group-based food frequency questionnaires (FG-FFQs), with a one-week (7-day FG-FFQ) and a one-month (30-day FG-FFQ) period of coverage for patients with pre-hypertension or hypertension. In 2010, 155 men and women, 30–70 years old, were invited to participate in a prospective study in two outpatient clinics in Porto Alegre, southern Brazil. The participants responded to two 30-day, two 7-day FG-FFQ, four 24-h dietary recalls, and underwent demographic, anthropometric, and blood pressure assessments. The validity and reproducibility were assessed using partial correlation coefficients adjusted for sex and age, and the internal validity was tested using the intra-class correlation coefficient. The participants were aged 61 (±10) years and 60% were women. The validity correlation coefficient was higher than r = 0.80 in the 30-day FG-FFQ for whole bread (r = 0.81) and the 7-day FG-FFQ for diet/light/zero soda and industrialized juices (r = 0.84) in comparison to the 24-h dietary recalls. The global internal validity was α = 0.59, but it increased to α = 0.76 when 19 redundant food groups were excluded. The reproducibility was higher than r = 0.80 for pasta, potatoes and manioc, bakery goods, sugar and cocoa, and beans for both versions. The 30-day had a slightly higher validity, both had good internal validity, and the 7-day FG-FFQ had a higher reproducibility.

## 1. Introduction

Hypertension is a risk factor for stroke and coronary heart disease, and other cardiovascular and non-cardiovascular diseases are among its consequences [1,2]. The challenge of preventing high blood pressure (BP) should drive population-based and clinical care interventions [2]. The increase in the prevalence of hypertension has occurred in low- and middle-income countries, despite rising detection and treatment rates [1,2,3]. The latter is based on the use of blood pressure lowering drugs; however, little attention has been paid to reducing blood pressure through lifestyle changes [2]. In clinical practice, counseling about a healthy diet, increasing physical activity [4,5,6,7], and quitting smoking should be added to blood pressure-lowering medication to control hypertension [4,8]. In recent decades, the Dietary Approach to Stop Hypertension (DASH) diet has been recommended to control blood pressure by increasing the intake of fruits, vegetables, and whole grains; changing to low-fat dairy foods and lean meat (preferably poultry and fish); and restricting salt consumption to 4 g per day [8]. The adherence to the DASH diet eating plan has been assessed in free-living individuals in prospective and experimental studies using DASH index punctuation-based metrics [9,10]. In the Nurses’ Health Study, a higher DASH index was associated with lower odds of a metabolically obese normal weight phenotype among younger participants [11] with a lower risk of long-term weight change [12] and a lower risk of cardiovascular disease and stroke among middle-aged women [12]. The adherence to the DASH diet predicts higher blood pressure reductions independently of weight loss; however, it may be sensitive to cultural aspects, requiring the need for counseling and monitoring strategies [10].

In addition to the DASH dietary score, several plant-based diet indices (PDIs), including the overall plant-based diet index (oPDI), the healthy PDI (hPDI), and the “Pro-Vegetarian Diet Index”, have been developed to positively score the intake of fruit, vegetables, whole grains, and nuts and legumes, and to penalize the consumption of animal products and weight differently the intake of alcohol, margarine, and dairy products [13]. Despite the similarities between the DASH diet and plant-based diets, a network meta-analysis ranked the DASH diet, out of 13 diets, as the most effective in reducing systolic and diastolic blood pressure among hypertensive and pre-hypertensive patients [14]. Therefore, we used the DASH diet to design a food group-based qualitative food frequency questionnaire (FG-QFFQ).

Diet records and 24-h dietary recalls are among the most commonly used methodologies to assess usual food and nutrient intake; however, these data collection methodologies are impracticable in clinical care and long-term follow-up studies. Alternatively, a food frequency questionnaire allows assessing the adherence to a dietary pattern and has lower complexity and operational cost [15,16]. However, implementing dietary counseling requires time and expertise, which makes it impossible for non-specialists to use it on a large scale. A methodological arsenal to address dietary changes does not have a simple instrument that can be used by health professionals in general, short enough to be incorporated into clinical practice and that does not require extensive data analysis. To overcome this gap, the FG-QFFQ was developed and validated to be used in two randomized clinic trials. Briefly, the first was the PREVER-prevention trial [17], a randomized placebo-controlled multicenter trial including participants with pre-hypertension, which assessed the effectiveness of low doses of blood pressure-lowering medications to prevent the incidence of hypertension. In a second study, the PREVER-treatment trial [18], two anti-hypertensive drugs were tested to control BP. In both trials, before the randomization, the participants underwent a lifestyle intervention including recommendations to consume a DASH-type diet and increase their physical activity. The present prospective study aimed to develop and assess the validity and reproducibility of a FG-QFFQ; the first assessed one week of food intake (7-day FG-QFFQ), and the second covered one month (30-day FG-QFFQ).

## 2. Materials and Methods

### 2.1. Study Participants

This cross-sectional study was conducted in 2010–2011 in Porto Alegre city, southern Brazil, including 155 men and women. The sample size definition was based on the recommendation of a minimum of 50 participants, preferably 100, and an average of 110 among food frequency questionnaires assessed worldwide [19]. The participants were selected from the outpatient clinic of hypertension and the primary healthcare center of the Hospital de Clínicas de Porto Alegre (HCPA). The eligibility criteria included individuals aged 30 to 70 years with mild hypertension or pre-hypertension who were invited to attend an appointment at the Clinical Research Center of the HCPA to assess blood pressure. The exclusion criteria included low life expectancy, previous cardiovascular disease (CVD), any kind of gastrointestinal disorder, pregnant women, or those who intended to become pregnant.

**Figure 1:**
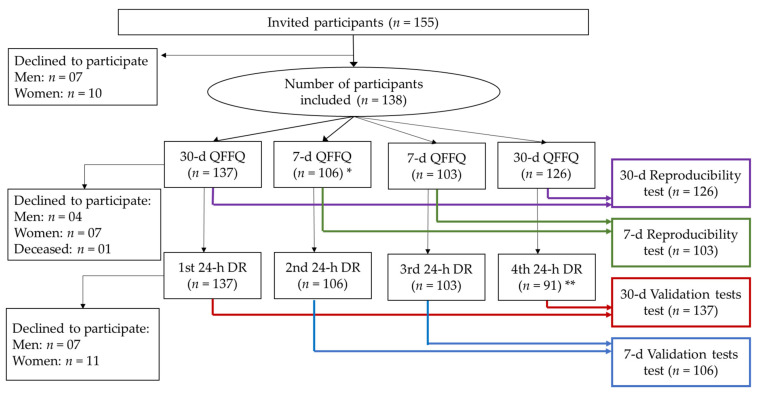
Participant selection and sequence of assessments flowchart. * The additional interview using the 7-day FG-QFFQ was initiated for the 28th participant onwards. Out of the 137 participants who initiated the study, 3 declined to continue in the second visit. ** The fourth interview using the 24-h dietary recall was initiated for the 28th participant onwards. Out of the 137 participants who initiated the study, 18 did not respond to the fourth interview.

The participants were evaluated four times within an interval of 7 to 14 days, providing information on the diet using the FG-QFFQ and health status (Figure 1). The multi-pass method was used to probe for food intake [20]. The identification of servings and portion sizes in the data collection using 24-h dietary recalls was supported by a photo album containing pictures of foods and serving sizes provided to the study participants to keep during the study [21,22].

### 2.2. Food Group-Based Food Frequency Questionnaire Design (FG-FFQ)

Two FG-QFFQs were administered by a health professional in person, assessing the intake of food items and food groups as the number of servings per day. The first assessed the dietary intake of the previous 30 days (30-day FG-QFFQ), and the second was a 7-day FG-QFFQ. The time frames were based on the empirical findings of the pilot study that suggested that a more accurate and productive assessment could be obtained with a shorter coverage period.

Both FG-FFQs had the same list of food items and food groups, which were created using data from the study of Syndrome of Obesity and Cardiovascular Risk Factors (SOFT study) [23,24,25]. The frequency questionnaire was previously validated in a randomly selected sample of the population of Porto Alegre [23]. The dietary data were validated using a semi-quantitative food frequency questionnaire compared to a 48-h dietary recall [25]. The details are described elsewhere [25,26, 27].

**Figure 2:**
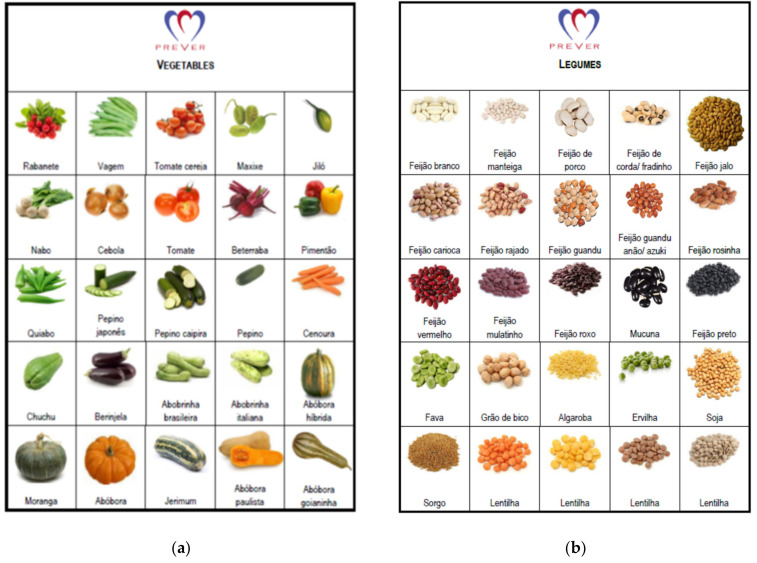

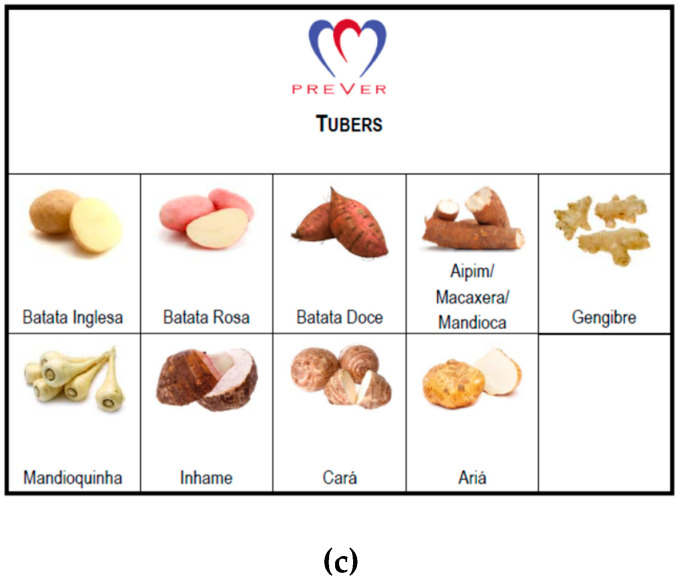
Vegetables, legumes, and tuber illustration cards. Schemes follow another format. Illustration used to help participants differentiating food groups used during the administration of the 30-day and 7-day FG-FFQs: (**a**) vegetables; (**b**) legumes; (**c**) tubers.

Food items and food groups recommended in the DASH diet eating plan were included in the food list and items or groups were discouraged by specialists to balance choices and the probability of risk exposure. Ultimately, the FG-QFFQ list consisted of 9 food items and 31 food groups. Each food group contained examples of items displayed in an illustrative catalog created for this study to help the participants to recollect (Figure 2). The food groups contained items selected from the 48-h dietary recall of the SOFT-Study [25] representing foods most commonly consumed in southern Brazil. To enlarge the coverage of the FG-FFQ to other states in Brazil and make them applicable to the PREVER trials, food items representative of other regions were included [28,29]. Both the 30-day and 7-day FG-FFQs aimed to assess the number of servings per day by asking: (1) how many times have you eaten (e.g., fruits) in the last 30 days?; (2) When have you eaten (e.g., fruits), how many servings, on average, have you eaten per day?

### 2.3. Diet Data Collection and Assessment

The food intake was assessed using the 30-day FG-QFFQ at the first and fourth visits, and the 7-day FG-QFFQ evaluated the intake at the second and third weeks. A 24-h dietary recall was applied in each of the four assessment sessions. We also assessed the time spent administering the 30-day FG-QFFQ, the 7-day FG-QFFQ, and the 24-h dietary recalls. In total, 137 participants responded to at least one of the 30-day FG-QFFQs and one of the 24-h dietary recalls, and 106 participants responded to the 7-day FG-QFFQ (Figure 1). The first 28 participants responded to the 30-day FG-QFFQ and 3 24-h dietary recalls, while 103 participants responded to 2 7-day FG-QFFQs, and 91 responded to the 4th 24-h dietary recall. Three participants had the assessment sessions rescheduled because of atypical food intake, increasing the average interval between evaluation sessions: one participant underwent a dental procedure, and two participants reported fasting for blood tests. Among the 138 participants, 91 completed all of the assessment sessions. The participants who declined to continue reported difficulties attending scheduled sessions, even by telephone. One participant died during the study.

The 24-h dietary recall protocol for data collection was based on the United States Department of Agriculture’s automated multiple-pass method [30]. The data from the 30-day and 7-day FG-QFFQs were entered into the Excel for Windows software for calculations, including doubled verification for inconsistencies. The data collected with 24-h dietary recalls were processed using the DietSys data system [31] and merged with the FG-QFFQs afterward. Handmade mixed dishes were broken down to calculate the intake of ingredients. The food items and ingredients were classified into food groups or items corresponding to the 40 items listed in the 30-day and the 7-day FG-QFFQs. For the data collected using the 24-h dietary recalls, the daily frequency of each food or ingredient intake was calculated according to Equation (1).

(1) $$FsGA_{ig}=\frac{\sum f_{igd}}{n_{d}}\;\times \;7$$

where $\sum f_{igd}$ is the sum of the number of servings per day (*f*) of each food group (*g*1, *g*2, *g*3, ... *g*40) of each participant (*i*1, *i*2, *i*3, ... *I*) per day (*d*1, *d*2, *d*3, *d*4); *n_d_* is the number of 24-h dietary recalls completed by each participant, multiplied by seven to calculate the number of servings per week.

The data collected using the 30-day FG-QFFQ were calculated using Equation (2), and for the 7-day FG-QFFQ, using Equation (3).

(2) $$Fs_{ig}=\frac{f_{ig}\;\times \;d_{ig}}{30.3}\;\times \;7$$

(3) $$Fs_{ig}=f_{ig}\;\times \;d_{ig}$$

where the number of servings per day (*d_ig_*) was multiplied by the number of days of each period (*f_ig_*) and by the number of servings per day (*d_ig_*). The result was divided by the number of days covered by each FG-QFFQ (30-day or 7-day). The frequency per week was equivalent to the number of servings consumed when a food or food group was consumed. The servings and portion sizes were not converted in weight and volume.

### 2.4. Non-Dietary Data Collection

In addition to dietary intake, we collected data on sex, age, education, height, and blood pressure at the first and fourth visits, monitoring the potential influence of the research inquiries on participant’s food choices. Standardized blood pressure [4] measurements were performed twice at each evaluation session (Figure 1), using an oscillometric monitor (OMRON HEM^®^–705 CP, Matsuzaka, Mie, Japan), and the average was used. The weight and height were also collected twice at each office visit, using internationally accepted standards [32,33], and the average was used. The study participants were asked to wear minimal clothes with no shoes to be weighed using a calibrated digital scale, with a capacity of 150 kg and precision of 100 g. Height was measured using an anthropometer, adhered to a wall free of baseboards, and measured with one centimeter. Body mass index (BMI) was calculated using weight in kilograms by height in meters squared [32].

### 2.5. Quality Control and Pilot Study

The questionnaires used in the data collection were administered by research assistants, certified before the initiation of data collection, and closely overseen by an experienced researcher. A pilot study was performed to test the standardized protocols and the feasibility of inquiring about the frequency of a food group’s intake. We enrolled 30 patients taking blood pressure-lowering medications who underwent the same procedures in the FG-QFFQ validation study. The findings from the pilot study led to the inclusion of three strategies to improve diet data quality.

(1) We generated a food catalog displaying illustrations of vegetables, tubers, and legumes (Figure 2), helping participants differentiate each food group. The catalog was used during the administration of the FG-QFFQs only.
(2) Examples of food items were added to the FG-QFFQ list to assist participants in remembering which items were part of each food group.
(3) Food items from other Brazilian regions were included as examples according to the guideline for regional food items produced by the Brazilian Ministry of Health [28] and the National Nutrition Survey conducted in Brazil [29], widening its applicability to the PREVER Study [17,18].

### 2.6. Statistical Analysis

We assessed three aspects of the validity and reproducibility of both FG-QFFQs: overall validity, internal validity, and reproducibility. The overall validity was tested using a partial correlation coefficient adjusted for sex and age, comparing the average intake of two 30-day and two 7-day FG-FFQs with the average intake of the four 24-h dietary recalls. The internal validity of the 30-day and the 7-day FG-QFFQ was tested, assessing the Cronbach’s alpha generated by the intra-class correlation coefficient. To assess the global internal validity, the target of the global Cronbach’s alpha was set at 0.70. To reach a global Cronbach’s alpha of 0.70, we excluded food items or food groups that had a Cronbach’s alpha below 0.01 until the point when the global Cronbach alpha was higher than 0.70. Four statistical models were performed until the target was reached. The reproducibility was tested using the Spearman correlation coefficient comparing the first and second 30-day FG-FFQ and 7-day FG-FFQ. The Bland–Altman method was used to determine whether there was a systematic difference between the 30-day FG-FFQ and the 7-day FG-FFQ compared to the 24-h dietary recall, and whether there was an acceptable agreement between the methods. The assessment of the plotting difference between the methods was compared against the average [34]. Statistical analyses were performed using the SPSS software.

## 3. Results

### 3.1. Demographic Characteristics and Interview

Table 1 shows the demographic characteristics of the participants, who were primarily women (60%) with a 1.7 kg/m^2^ higher BMI than the men and a lower education level (2.1 years of study). The average systolic and diastolic blood pressure were similar between men and women. The BMI and systolic and diastolic blood pressure were stable over the study period.

Table 2 shows the time spent administering the 30-day and 7-day FG-QFFQs. In the entire group of participants, it took four more minutes to complete the 30-day FG-QFFQ than the 7-day FG-QFFQ (*p* < 0.001). The mean difference of time spent to administer the 30-day in comparison to the 7-day FG-QFFQ was 5 min for women and 3 min for men (*p* < 0.001).

### 3.2. Food Intake Assessment

Table 3 displays the mean and standard deviation of the dietary intake assessed with the 30-day FG-QFFQ, the 7-day FG-QFFQ, and the 24-h dietary recall in the number of servings consumed per week. The frequency of bakery goods and fast foods was negligible per day.

When comparing the food intake assessed with the 30-day FG-QFFQ, the 7-day FG-QFFQ, and the 24-h dietary recall, there were negligible differences between the intake of meats, whole rice, refined biscuit and cracker, whole biscuit and cracker, pasta, bakery goods, fast food, nuts, legumes, dairy products, pickles, and fried foods.

### 3.3. Overall Validity

Table 4 displays the overall validity of FG-QFFQs compared to the 24-h dietary recalls, tested using a partial correlation coefficient adjusted for sex and age.

The average correlation between the FG-FFQ and the 24-h dietary recall was 0.45 for both the 30-day and the 7-day FG-FFQ, but it was not similar for all of the items. In the 30-day FG-QFFQ, the correlations were higher than 0.70 for processed meat (r = 0.72); white bread, cake, and sweet bread (r = 0.78); whole bread (r = 0.81), light cheese, cream, and cream cheese (r = 0.76); and animal-based fat and salted margarine (r = 0.74). The correlation between the intake of white, whole bread, and animal fat food groups was lower in the 7-day FG-QFFQ (r = 0.55, r = 0.62, and 0.66, respectively). Moderate correlations (from 0.40 to 0.69) were found for most food items in both the 30-day and 7-day FG-QFFQ, such as sugar and cocoa, sweets, bovine meat without visible fat, white rice, brown rice, refined biscuit or cracker, whole biscuit, or cracker, regular soda and industrialized juices, nuts, light or diet yogurt, whole and semi-skim milk, regular cheese, cream, and cream cheese, fried foods, vegetable fat and unsalted margarine, fruits, and leafy vegetables. For the data collected with the 7-day FG-QFFQ, a moderate correlation was found for pasta, bread, cake, sweetbread, beans, whole yogurt, pickles, animal fat and unsalted margarine, fruits, and fresh fruit juices. Lower correlations were found for bovine meat with visible fat, chicken with skin, chicken without skin, other meat, fish, shrimp, seafood, pasta (only for the 30-day FG-QFFQ), salty industrialized sauces and soups, regular soda and industrialized juices, bakery goods, fast foods, beans (in the 30-day FG-QFFQ only), legumes, whole yogurt (in the 30-day FG-QFFQ only), skim milk, pickles (in the 30-day FG-QFFQ only), vegetable fat and salty margarine (in the 7-day FG-QFFQ only), potatoes and manioc, fresh fruit juices (in the 30-day FG-QFFQ only), vegetables, and leafy vegetables (in the 7-day FG-QFFQ only).

### 3.4. Internal Validity

Table 5 shows the internal validity of the two FG-QFFQs. We tested the global internal validity of each FG-QFFQ and the individual items to assess for redundancy.

When a food item contributed in less than 0.01 to the global internal validity, it was excluded. For the 30-day and 7-day FG-QFFQ, chicken meat with skin, whole rice, refined biscuit and cracker, whole biscuit and cracker, whole bread, light/diet yogurt, skim milk, light cheese, cream, and cream cheese were removed because they did not reach the Cronbach’s alpha of 0.01 in one of the four statistical models; therefore, they were excluded from both of the FG-QFFQs. In the 30-day FG-QFFQ, the intake of fish, shrimp, and seafood, nuts, whole yogurt, fruits, fresh fruit juices, vegetables, and leafy vegetables contributed very little to the global internal validity; therefore, they were excluded. In the 7-day FG-QFFQ, unhealthy food items had a lower Cronbach’s alpha. They could be removed to improve the global internal validity (e.g., chicken meat with skin, other meats, salty industrialized sauces and soups, regular and diet/light/zero soda and industrialized juices, bakery goods, fast foods, pickles, fried foods, and animal fat and salty margarine).

### 3.5. Reproducibility

**Figure 3:**
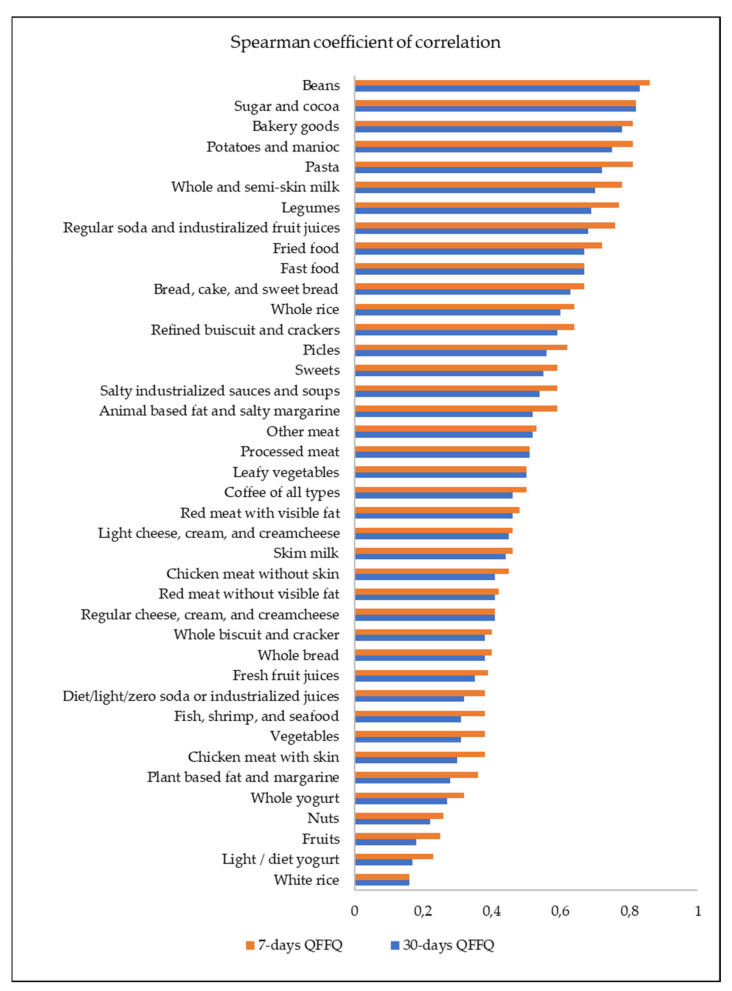
Spearman coefficient of correlation between the first and the second 30-day FG-QFFQ and 7-day FG-QFFQ used to test the reproducibility of measures; *p*-value < 0.01.

The average reproducibility was 0.49 and 0.53 for the 30-day and the 7-day FG-FFQ, respectively, and correlation coefficients were statistically significant (Figure 3).

White rice (r = 0.16); regular cheese, cream, and cream cheese (r = 0.41); leafy vegetables (r = 0.50); processed meat (r = 0.51); fast food (r = 0.67); and sugar and cocoa (r = 0.82) had equal coefficients of correlation between the first and second tests for the 7-day and the 30-day FG-FFQ. Both of the FG-FFQs were highly reproducible in assessing beans (r = 0.83), sugar, and cocoa (r = 0.82). Thirteen out of forty food items and food groups had a correlation coefficient below r = 0.40 (e.g., white rice, fruits, whole bread, light/diet yogurt, and whole yogurt). In general, the food items and food groups had a close correlation coefficient for the 30-day and 7-day FG-FFQ, indicating that both had good reproducibility; however, the 7-day FG-FFQ reached higher correlations.

### 3.6. Agreement

**Figure 4:**
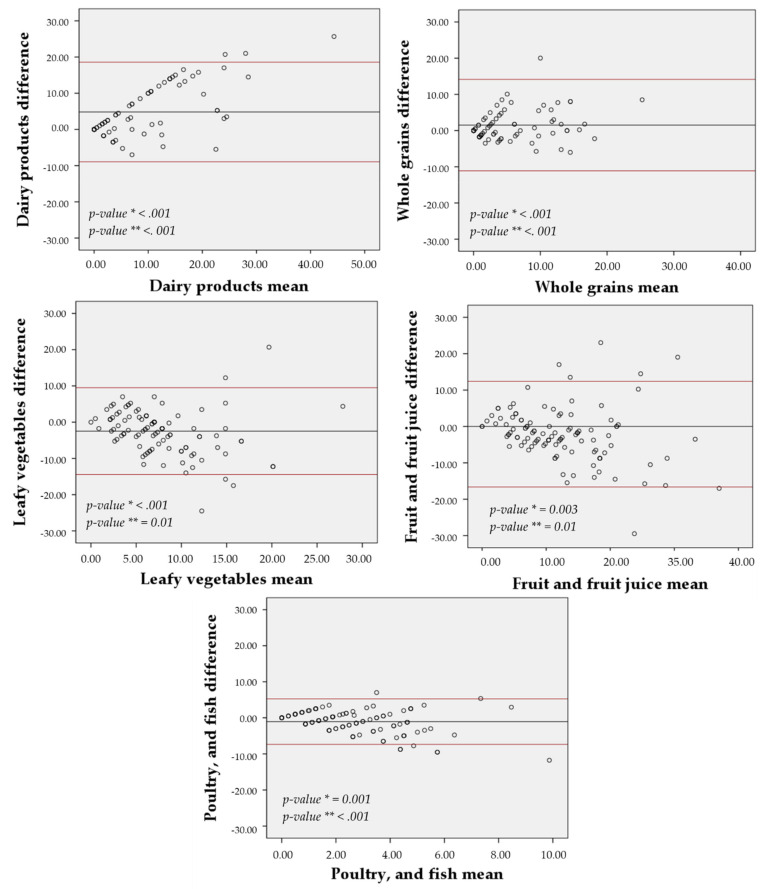
Bland-Altman agreement between the 30-day FG-QFFQ with the 24-h dietary recalls. * *p*-value for non-zero difference between methods (*t*-student test) and ** *p*-value for propensity bias (regression model).

**Figure 5:**
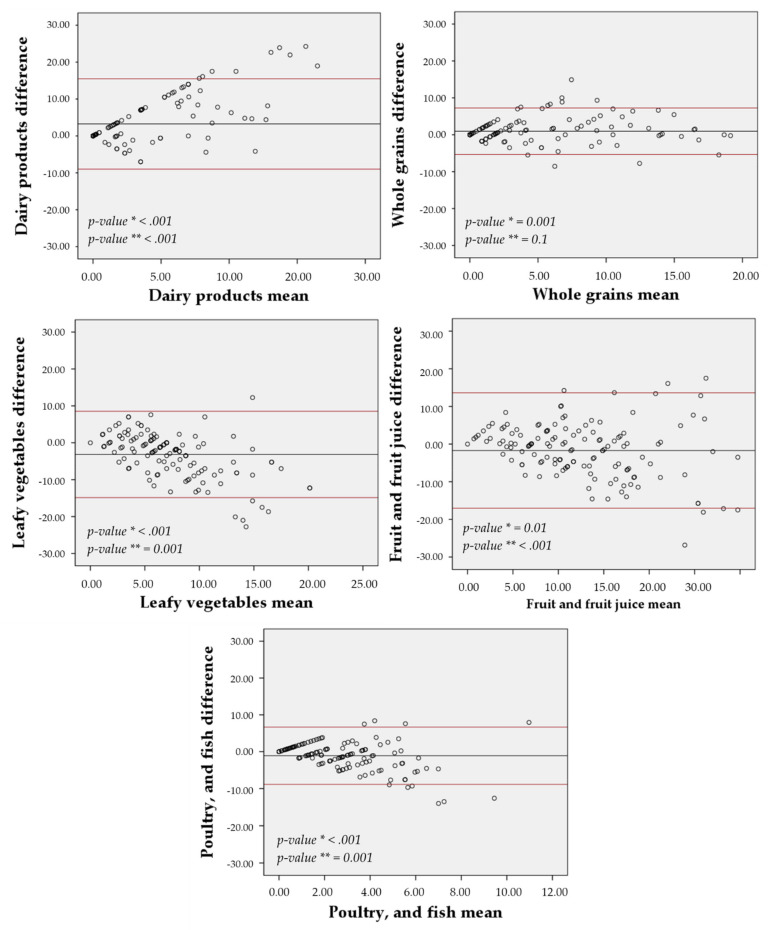
Bland-Altman agreement between the 7-day FG-QFFQ with the 24-h dietary recalls. * *p*-value for non-zero difference between methods (*t*-student test) and ** *p*-value for propensity bias (regression model).

The agreement between the 7-day and the 30-day FG-FFQ with the 24-h dietary recall was displayed in Figure 4 and Figure 5, focusing on the five most food groups most advocated in the DASH diet eating plan. The food frequency of weekly mean difference of whole grains (30-day FG-FFQ = 0.96; 7-day FG-FFQ = 1.52), fruits and fruit juices (30-day FG-FFQ = −1.71; 7-day FG-FFQ = −2.22), and poultry and fish (30-day FG-FFQ = −1.10; 7-day FG-FFQ = −1.07) was closer to zero in both of the FG-FFQs, but the difference was larger for dairy products (30-day FG-FFQ = 3.26; 7-day FG-FFQ = 4.82) and vegetables (30-day FG-FFQ = −3.16; 7-day FG-FFQ = 2.48). The intake of whole grains using the 7-day and the 30-day FG-FFQ showed the greater graphical agreement with the 24-h dietary recall, with a closer to zero weekly food intake difference and lower data dispersion around the average, and fruits and fruit juices presented the poorer agreement. However, all food groups had an acceptable agreement with most of the values’ dispersion covered by the 95% confidence interval.

## 4. Discussion

The 30-day and 7-day FG-FFQ, comprising 9 food items and 31 food groups, were easily applicable, valid, and reproducible, informing the patient’s daily number of foods servings and food group’s consumption pre-hypertension or hypertension under treatment.

### 4.1. The Pilot Study and the FG-FFQ Design

Food frequency questionnaires (FFQs) were designed to assess general and specific aspects of diet [16,35]. The pilot study made an empirical assessment of three main aspects of the food frequency questionnaires’ feasibility and ultimately developed an FG-FFQ adequate to a large study population. The cultural context of an FFQ depends on awareness, sensitivity, appropriateness, and competency [35]. These cultural aspects of the FG-FFQ included the report’s accuracy of serving sizes since it relies on cognitive capacity [35]. Second, cultural sensitivity and appropriateness driven by the FFQ should accommodate food items according to cultural behaviors in inoffensive ways. Both definitions were reached by including examples of foods locally consumed and in other regions of Brazil [28,29] and by using the food examples catalog (Figure 2), preventing study participants from feeling disregarded about items consumed by cultural groups. Third, the inadequacy of a culturally incompetent approach was taken into account by grouping food items according to the DASH diet eating plan (e.g., skim milk and chicken without skin) and according to unrecompensed food groups (soda, salty industrialized foods) [35].

The development of both of the FG-FFQs also considered objective aspects of the diet. Forty-six percent of food frequency questionnaires validated worldwide use data collected previously in the target population to guide the list of foods construction [35]. To expand the coverage of the food items and food groups, allowing the use of FG-FFQ in other regions of Brazil, we listed food items based on national information [28] and the Brazilian Ministry of Health guideline that described regional foods [29]. Some items, such as coffee of all types, rice, whole rice, beans, and pasta, were kept ungrouped because they represent a large proportion of the Brazilian population’s diet [29], while foods such as *jiló* were cited within food groups, assisting the participants to remember foods that were commonly consumed only in the north and northeast regions [29].

We used photographs to stimulate the participants’ memory and the DASH diet assessment approach to access the food group-based design of the FG-FFQs inspired by a web-based FFQ developed in the United States [36]. The 30-day and 7-day FG-FFQ were also used to group food items with nutritional similarities but different portions or serving sizes. Therefore, the selection of serving sizes was removed from the FG-FFQ form because, in the pilot study, participants could not choose one serving size to represent a food group (e.g., fruits: grapes in a bunch of individual units in comparison to oranges). In 1984, Samet and colleagues assessed food frequency interviews to preformed vitamin A and beta-carotene in an ongoing population-based case-control study of lung cancer. They concluded that the frequency alone was an appropriate measure when the objective of the data collection is to assess a specific nutrient [37].

Shorter lists of foods and the omission of serving sizes expedite dietary assessment without affecting the reproducibility and the food pattern estimation [36]. Polish researchers stated that the long time of application was the main difficulty imposed by the long list and foods detailing of the semi-quantitative food frequency questionnaire validated by them [34]. To overcome this limitation, Niedzwiedzka and colleagues proposed simplifying the food frequency questionnaires, shortening the list of items by grouping foods, and omitting servings or portion sizes [36]. As a result, a reproducible, non-quantitative 62-item food frequency questionnaire was developed to assess the general food intake and food patterns in the young Polish female population [36].

In the present study, both of the FG-FFQs were applied in a short time interview, ranging from 15 to 20 min each on average. There was no significant difference in time between men and women during the interview; however, the 7-day FG-FFQ was 4 min faster than the 30-day FG-FFQ. As discussed by Martela and colleagues, the clinical nutrition practice routine makes it possible to run a full analysis and diagnosis of the nutritional status and food and nutrient assessment that might be performed in one or more visits. However, this full assessment requires experience and availability of time from both the patient and health professional [38]. Such conditions are not usually feasible in the outpatient clinic routine because a health professional usually conducts the interview within a very limited time.

### 4.2. Validity

Using food groups and inquiring only about the frequency of consumption expedited the dietary intake assessment without causing flaw to the accuracy of most food groups; so many of the items in the 30-day and 7-day FG-FFQ had the same average correlation. This aspect of the FG-FFQs was corroborated by other studies that proposed FFQs with a reduced number of food items to assess the dietary intake in specific health conditions [39,40]. In our study, the list included nine ungrouped foods to cover food items that primarily represent the Brazilian population’s food intake [29]. Most of these foods reached moderate or robust correlation coefficients [41] between both 30-day and 7-day FG-FFQs with the 24-h dietary recalls but had a lower correlation when food items were of difficult differentiation by the participants (e.g., milk and skim milk), as it has also been found in a similar validation study [40]. In the same study, rice, pasta, and wheat were grouped, and the researchers found a fair correlation validity [40].

Similarly, we also found a fair correlation for white rice and pasta in the 30-day FG-FFQ and the 7-day FG-FFQ, even inquiring as a separate food. Separating whole from fat-reduced dairy foods was important to comply with the DASH diet plan that advocates favoring fat-reduced dairy products [5]. It is important to highlight those food items, to which closer attention from the study participants to describe and consider the details, can have the validity harmed because of cultural awareness [35].

Most of the food groups reached acceptable validity in comparison to other studies. Bredin et al. assessed the validity of a short FFQ developed to assess non-alcoholic fatty liver disease patients [39]. Similar to our study, the intake of fruits and vegetables was of great importance due to the study participants’ health condition, and both reached fair correlations [39].

However, some aspects of both of the FG-FFQs deserve mention. Neither FG-FFQ could assess the intake of fast-food items, probably due to the cost of such items. Moreover, in the overall internal validity assessment, two groups of chicken with and without skin and red meat with or without visible fat did not add accuracy and were redundant, affecting the reported consumption in the 7-day FG-FFQ. Food items used as ingredients, such as animal-based fat, plant-based fat, salty margarine, and sugar, were also barely captured by either FG-FFQ, and this was a result of the probing techniques used in the 24-h dietary recall that do not apply to the FFQs [30].

### 4.3. Reproducibility

In general, the 7-day FG-QFFQ had better reproducibility than the 30-day FG-QFFQ, although their difference was negligible. In the participants assessed using the 30-day FG-QFFQ, 13 food items had fair reproducibility, and 6 strongly correlated. These results were similar to the findings of other studies assessing the reproducibility of food frequency questionnaires developed for a population with specific health conditions [40,42,43]. These food items included white rice that had acceptable validity but very low reproducibility. An opposite result was found for pasta and bakery goods, suggesting that a report can be accurate because it correlates with the true food intake while not reproducible by FFQs. This finding is corroborated by results found in other simplified FFQs [36,40,43].

### 4.4. Strengths

In clinical practice, the operational recommendations for changing the diet and assessing the degree of adherence usually require time and some level of expertise. While there are various methods for dietary assessment, a simplified method could streamline the process if it could distinguish recommendations that should be reinforced from those that have been adopted. Our FG-QFFQ can facilitate dietary change counseling because it identifies recommendations that require more attention from the healthcare professional. This agility allows the recommendations to be made as part of the consultation and even without nutrient or energy intake information. It also allows the evaluation to occur over short periods (7 days) or with a longer duration (30 days).

### 4.5. Limitations

This study aimed to determine the validity and reproducibility of food group intake, but the FG-QFFQ does not provide the average intake of food items or specific nutrient content. However, the food group-based food frequency questionnaire quantifies the number of servings. Therefore, there was no adjustment for energy intake in the correlations tested in this study [16], which could be considered a potential limitation.

## 5. Conclusions

The 30-day and the 7-day FG-FFQ had acceptable global and internal validity and reproducibility, and both were applicable in a busy outpatient clinic where there is little time for interviews. Moreover, the 7-day FG-FFQ was applied in the run-in of the PREVER study, and it has been proven to be applicable for monitoring diet intake. Nevertheless, it is essential to consider those few food items that had a poor correlation owing to cultural awareness, and those same food items might have different levels of validity and reproducibility.

## Figures and Tables

**Table 1:** Characteristics of the participants. Means ± standard deviation.

	Overall (*n* = 137)	Men (*n* = 55)	Women (*n* = 82)	*p*-Value *
Age	61 ± 10	63 ± 11	61 ± 9	0.2
Education, in years of study	8.6 ± 4.0	9.9 ± 4.2	7.8 ± 3.6	0.07
Body mass index (kg/m^2^)	30.5 ± 5.6	29.5 ± 4.8	31.2 ± 6.1	0.09
First visit				
Systolic blood pressure (mm Hg)	137.8 ± 18.9	139.2 ± 19.3	136.9 ± 18.7	0.5
Diastolic blood pressure (mm Hg)	82.4 ± 12	82 ± 11.7	82.6 ± 12.3	0.8
Body mass index (kg/m^2^)	30.5 ± 5.6	29.4 ± 4.8	31.2 ± 6	0.08
Fourth Visit				
Systolic blood pressure (mm Hg)	135.3 ± 21.8	137.3 ± 20.6	134 ± 22.7	0.4
Diastolic blood pressure (mm Hg)	80.1 ± 12.8	80.8 ± 13.8	79.6 ± 12.1	0.6
Body mass index (kg/m^2^)	30.2 ± 5.6	29 ± 4.1	31 ± 6.3	0.06

* *p*-value for the test of the difference between men’s and women’s averages.

**Table 2:** Average time spent in the dietary assessment procedures for the 30-day FG-QFFQ, the 7-day FG-QFFQ, and the 24-h dietary recall.

	Overall (*n* = 137)	Men (*n* = 55)	Women (*n* = 82)	*p*-Value^1^
Time Spent to Assess Dietary Intake in Minutes [Mean ± SD]				
30-day FG-QFFQ ^2^	0:20 ± 0:07	0:21 ± 0:09	0:20 ± 0:06	0.7
7-day FG-QFFQ ^2^	0:15 ± 0:05	0:15 ± 0:06	0:15 ± 0:04	0.7
Mean difference ^2,3^	0:04 ± 0:08	0:05 ± 0:08	0:03 ± 0:06	<0.001
24-h dietary recall	0:19 ± 0:05	0:19 ± 0:06	0:19 ± 0:04	0.5
First visit				
30-day FG-QFFQ	0:21 ± 0:08	0:21 ± 0:10	0:22 ± 0:07	0.7
24-h dietary recall	0:19 ± 0:06	0:20 ± 0:08	0:18 ± 0:05	0.09
Second Visit				
FG-QFFQ 7-days	0:15 ± 0:05	0:14 ± 0:04	0:16 ± 0:06	0.07
24-h dietary recall	0:20 ± 0:09	0:21 ± 0:09	0:20 ± 0:09	0.6
Third visit				
7-day FG-QFFQ	0:15 ± 0:08	0:16 ± 0:11	0:15 ± 0:05	0.4
24-h dietary recall	0:19 ± 0:08	0:19 ± 0:08	0:18 ± 0:07	0.5
Fourth visits				
30-day FG-QFFQ	0:19 ± 0:08	0:20 ± 0:10	0:18 ± 0:06	0.1
24-h dietary recall	0:18 ± 0:07	0:17 ± 0:06	0:19 ± 0:07	0.1

FG-QFFQ = Food groups-based qualitative food frequency questionnaire; ^1^ *p*-value for the test of the difference between men’s and women’s averages; ^2^ Average of the 30-day FG-QFFQ, 7-day FG-QFFQ, and the 24-h dietary recalls; ^3^ Mean difference between the 30-day FG-QFFQ and the 7-day FG-QFFQ.

**Table 3:** The mean and standard deviation of food items and food groups intake assessed using the 30-day FG-QFFQ, the 7-day FG-QFFQ, and the 24-h dietary recall.

Food Items and Food Groups (Servings per Week)	Mean ± SD		
	30-Day FG-QFFQ (*n* = 137)	7-Day FG-QFFQ (*n* = 106)	24-h DR (*n* = 137)
Sugar and cocoa	5.3 ± 6.5	4.1 ± 5.6	13.9 ± 10
Coffee of all types	12.4 ± 7.7	12.1 ± 7.3	12.7 ± 6.3
Sweets	4.3 ± 4.3	4.2 ± 4.9	6.6 ± 6.2
Red meat with visible fat	1.7 ± 1.9	1.5 ± 2.3	0.8 ± 1.4
Red meat without visible fat	1.9 ± 1.9	2.3 ± 3.2	4.1 ± 3.3
Chicken meat without skin	1.0 ± 1.4	1.2 ± 1.5	1.0 ± 1.7
Chicken meat with skin	1.5 ± 1.9	1.4 ± 1.7	2.7 ± 2.9
Processed meat	3.3 ± 3.8	3.6 ± 4.0	3.7 ± 4.2
Other meat	0.5 ± 0.6	0.5 ± 0.9	2.5 ± 2.8
Fish, shrimp, and seafood	0.6 ± 0.7	0.6 ± 0.9	0.5 ± 1.2
White rice	6.8 ± 3.6	6.9 ± 4.8	6.1 ± 3.2
Whole rice	0.6 ± 1.4	0.6 ± 1.5	0.4 ± 1.3
Refines biscuit and cracker	1.4 ± 2.9	1.3 ± 2.7	1.5 ± 2.8
Whole biscuit and cracker	0.4 ± 0.9	0.5 ± 1.5	0.4 ± 1.1
Pasta	1.3 ± 1.1	1.3 ± 1.4	2.1 ± 2.3
Bread, cake, and sweet bread	7.3 ± 5.1	8.0 ± 7.6	8.1 ± 5.5
Whole bread	3.8 ± 4.7	4.1 ± 7.5	2.9 ± 4.4
Salty industrialized sauces and soups	2.2 ± 2.8	2.3 ± 3.0	6.1 ± 4.5
Regular soda and industrialized juices	2.6 ± 3.7	2.8 ± 4.1	3.5 ± 4.5
Diet/light/zero soda and industrialized juices	1.3 ± 3.0	1.9 ± 4.1	1.6 ± 3.1
Bakery goods	0.2 ± 0.4	0.2 ± 0.4	0.2 ± 0.8
Fast food	0.3 ± 0.8	0.2 ± 0.4	0.4 ± 1.4
Nuts	1.0 ± 1.9	1.0 ± 2.2	0.4 ± 1.2
Beans	5.4 ± 4.2	5.1 ± 3.4	3.8 ± 2.9
Legumes	0.7 ± 1.0	0.6 ± 1.1	0.1 ± 0.5
Light/diet yogurt	0.4 ± 1.7	0.7 ± 3.7	0.6 ± 1.7
Whole yogurt	0.9 ± 2.1	0.6 ± 1.5	0.1 ± 0.5
Skim milk	3.3 ± 5.5	3.3 ± 5.2	0.2 ± 1.3
Whole and semi-skim milk	4.9 ± 6.0	5.0 ± 6.8	10.8 ± 7.2
Light cheese, cream, and cream cheese	1.0 ± 2.4	0.9 ± 2.3	0.7 ± 1.7
Regular cheese, cream, and cream cheese	3.5 ± 3.7	4.0 ± 5.0	5.4 ± 5.1
Pickles	0.6 ± 1.4	0.5 ± 0.9	1.0 ± 2.1
Fried foods	0.8 ± 1.0	0.8 ± 0.9	0.7 ± 1.4
Animal-based fat and salty margarine	2.4 ± 3.8	2.2 ± 3.8	25 ± 9.5
Plant-based fat and salty margarine	11.1 ± 5.7	13.1 ± 9.8	5.4 ± 5.0
Potatoes and manioc	1.8 ± 1.6	1.6 ± 1.8	2.9 ± 2.8
Fruits	9.6 ± 5.1	9.4 ± 6.2	12.6 ± 8.6
Fresh fruit juices	5.3 ± 3.1	5.4 ± 3.7	0.9 ± 1.8
Vegetables	2.2 ± 3.7	1.9 ± 3.5	9.0 ± 6.3
Leafy vegetables	5.8 ± 3.4	6.5 ± 5.0	11.9 ± 6.7

**Table 4:** Partial correlation coefficient (r) comparing the 30-day FG-QFFQ and the 7-day FG-QFFQ with the 24-h dietary recall adjusted for age and gender.

Food Items and Food Groups (Servings per Week)	30-Day FG-QFFQ	*p*-Value	7-Day FG-QFFQ	*p*-Value
Sugar and cocoa	0.51	<0.001	0.52	<0.001
Coffee of all types	0.69	<0.001	0.73	<0.001
Sweets	0.51	<0.001	0.43	<0.001
Red meat with visible fat	0.22	0.009	0.10	0.315
Red meat without visible fat	0.41	<0.001	0.43	<0.001
Chicken meat without skin	0.37	<0.001	0.17	0.074
Chicken meat with skin	0.13	0.1	0.23	0.018
Processed meat	0.72	<0.001	0.70	<0.001
Other meat	0.29	0.001	0.13	0.196
Fish, shrimp, and seafood	0.18	0.03	0.13	0.188
White rice	0.57	<0.001	0.57	<0.001
Whole rice	0.68	<0.001	0.35	<0.001
Refines biscuit and cracker	0.55	<0.001	0.62	<0.001
Whole biscuit and cracker	0.62	<0.001	0.67	<0.001
Pasta	0.24	0.005	0.45	<0.001
Bread, cake, and sweet bread	0.78	<0.001	0.55	<0.001
Whole bread	0.81	<0.001	0.62	<0.001
Salty industrialized sauces and soups	0.32	<0.001	0.33	0.001
Regular soda and industrialized juices	0.6	<0.001	0.34	<0.001
Diet/light/zero soda and industrialized juices	0.76	<0.001	0.84	<0.001
Bakery goods	0.24	0.005	0.29	0.002
Fast food	0.15	0.08	0.16	0.108
Nuts	0.66	<0.001	0.78	<0.001
Beans	0.35	<0.001	0.50	<0.001
Legumes	0.11	0.2	0.34	<0.001
Light/diet yogurt	0.56	<0.001	0.57	<0.001
Whole yogurt	0.09	0.3	0.48	<0.001
Skim milk	0.23	0.007	0.29	0.002
Whole and semi-skim milk	0.55	<0.001	0.49	<0.001
Light cheese, cream, and cream cheese	0.76	<0.001	0.74	<0.001
Regular cheese, cream, and cream cheese	0.66	<0.001	0.57	<0.001
Pickles	0.21	0.02	0.45	<0.001
Fried foods	0.45	<0.001	0.41	<0.001
Animal-based fat and salty margarine	0.74	<0.001	0.66	<0.001
Plant-based fat and salty margarine	0.46	<0.001	0.29	0.002
Potatoes and manioc	0.26	0.003	0.29	0.002
Fruits	0.54	<0.001	0.57	<0.001
Fresh fruit juices	0.38	<0.001	0.55	<0.001
Vegetables	0.22	0.01	0.36	<0.001
Leafy vegetables	0.57	<0.001	0.36	<0.001

**Table 5:** Internal validity of the 30-day and 7-day FG-QFFQ using the intra-class confidence interval to estimate Cronbach’s alpha.

Food Items	30-Day FG-QFFQ ^1^				7-Day FG-QFFQ ^1^			
	Initial Model		Final Model		Initial Model		Final Model	
	R	A	r	α	r	A	r	α
Global internal validity		0.59		0.74		0.61		0.76
Sugar and cocoa ^5^	0.14	0.53	0.32	0.65	−0.07	0.65	-	-
Coffee of all types	0.31	0.49	0.23	0.67	0.33	0.60	0.25	0.74
Sweets	0.28	0.51	0.24	0.65	0.33	0.60	0.28	0.74
Red meat with visible fat	0.16	0.53	0.28	0.65	0.41	0.61	0.46	0.73
Red meat without visible fat	0.23	0.52	0.24	0.66	0.17	0.62	0.25	0.74
Chicken meat without skin ^7^	0.10	0.53	0.20	0.66	0.39	0.62	-	-
Chicken meat with skin ^2,6^	−0.01	0.54	-	-	0.05	0.63	-	-
Processed meat ^6^	0.32	0.50	0.35	0.64	0.15	0.62	0.13	0.75
Other meat	0.07	0.53	0.13	0.66	0.03	0.63	-	-
Fish, shrimp, and seafood ^2^	−0.05	0.54	-	-	0.35	0.62	0.38	0.74
White rice	0.24	0.51	0.37	0.64	0.39	0.60	0.49	0.72
Whole rice ^2,6^	−0.03	0.54	-	-	0.02	0.63	-	-
Refines biscuit and cracker ^3,5^	0.07	0.53	-	-	−0.04	0.63	-	-
Whole biscuit and cracker ^3,5^	0.09	0.53	-	-	−0.03	0.63	-	-
Pasta	0.07	0.53	0.18	0.66	0.24	0.62	0.20	0.74
Bread, cake, and sweet bread	0.23	0.51	0.46	0.62	0.46	0.58	0.61	0.70
Whole bread ^1,6^	−0.12	0.56	-	-	0.03	0.64	-	-
Salty industrialized sauces and soups ^5^	0.11	0.53	0.17	0.66	−0.03	0.63	-	-
Regular soda and industrialized juices	0.19	0.52	0.36	0.64	0.00	0.63	-	-
Diet/light/zero soda and industrialized juices	0.15	0.52	0.13	0.66	0.03	0.63	-	-
Bakery goods	0.06	0.53	0.18	0.66	0.00	0.63	-	-
Fast food ^6^	0.20	0.53	0.20	0.66	−0.04	0.63	-	-
Nuts ^3^	0.06	0.53	-	-	0.13	0.62	0.11	0.74
Beans	0.14	0.52	0.19	0.66	0.11	0.62	0.14	0.74
Legumes	0.24	0.53	0.09	0.66	0.13	0.62	0.14	0.74
Light/diet yogurt ^2,5^	−0.05	0.54	-	-	−0.05	0.64	-	-
Whole yogurt ^3^	0.06	0.53	-	-	0.17	0.62	0.14	0.74
Skim milk ^2,5^	−0.08	0.56	-	-	−0.08	0.64	-	-
Whole and semi-skim milk	0.20	0.52	0.32	0.64	0.34	0.60	0.40	0.73
Light cheese, cream, and cream cheese ^3,6^	0.04	0.53	-	-	0.05	0.63	-	-
Regular cheese, cream, and cream cheese	0.23	0.51	0.20	0.66	0.33	0.60	0.38	0.73
Pickles ^5^	0.33	0.52	0.32	0.66	−0.10	0.63	-	-
Fried foods ^5,6^	0.21	0.53	0.36	0.66	0.09	0.63	-	-
Animal-based fat and salty margarine ^5^	0.06	0.53	0.18	0.66	−0.11	0.64	-	-
Plant-based fat and salty margarine	0.22	0.51	0.22	0.66	0.41	0.58	0.46	0.73
Potatoes and manioc	0.21	0.52	0.28	0.66	0.28	0.62	0.31	0.74
Fruits ^3^	0.14	0.53	-	-	0.34	0.60	0.39	0.73
Fresh fruit juices ^4^	0.28	0.51	-	-	0.39	0.60	0.40	0.73
Vegetables ^3^	0.13	0.53	-	-	0.10	0.62	0.13	0.74
Leafy vegetables ^3^	0.02	0.54	-	-	0.46	0.59	0.52	0.72

^1^ To reach the targeted global Cronbach’s alpha ≥0.7 in the internal validity modeling of the FG-QFFQ, food items with the individual Cronbach’s alpha <0.01 were excluded. Four statistical models were tested to reach this goal; ^2^ Food items or food groups were excluded in the second model in the 30-day FG-QFFQ; ^3^ Food items or food groups were excluded in the third model in the 30-day FG-QFFQ; ^4^ Food items or food groups were excluded in the fourth model in the 30-day FG-QFFQ; ^5^ Food items or food groups were excluded in the second model in the 7-day FG-QFFQ; ^6^ Food items or food groups were excluded in the third model in the 7-day FG-QFFQ; ^7^ Food items or food groups were excluded in the fourth model in the 7-day FG-QFFQ.

## Data Availability

The data presented in this study are available on request from the corresponding author.

## References

[1] Zhou B., Carrillo-Larco R.M., Danaei G., Riley L.M., Paciorek C.J., Stevens G.A., Gregg E.W., Bennett J.E., Solomon B., Singleton R.K. (2021). Worldwide trends in hypertension prevalence and progress in treatment and control from 1990 to 2019: A pooled analysis of 1201 population-representative studies with 104 million participants. Lancet.

[2] Picon R.V., Fuchs F.D., Riegel G., Fuchs S.C. (2012). Trends in prevalence of hypertension in Brazil: A systematic review with meta-analysis. PLoS ONE.

[3] Olsen M.H., Angell S.Y., Asma S., Boutouyrie P., Burger D., A Chirinos J., Damasceno A., Delles C., Gimenez-Roqueplo A.-P., Hering D. (2016). A call to action and a lifecourse strategy to address the global burden of raised blood pressure on current and future generations: The Lancet Commission on hypertension. Lancet.

[4] Sacks F.M., Svetkey L.P., Vollmer W.M., Appel L.J., Bray G.A., Harsha D., Obarzanek E., Conlin P.R., Miller E.R., Simons-Morton D.G. (2001). Effects on Blood Pressure of Reduced Dietary Sodium and the Dietary Approaches to Stop Hypertension (DASH) Diet. N. Engl. J. Med..

[5] Appel L.J., Moore T.J., Obarzanek E., Vollmer W.M., Svetkey L.P., Sacks F.M., Bray G.A., Vogt T.M., Cutler J.A., Windhauser M.M. (1997). A Clinical Trial of the Effects of Dietary Patterns on Blood Pressure. N. Engl. J. Med..

[6] Guilbert J.J. (2003). The world health report 2002-Reducing risks, promoting healthy life. Educ. Health.

[7] Chobanian A.V., Bakris G.L., Black H.R., Cushman W.C., Green L.A., Izzo J.L., Jones D.W., Materson B.J., Oparil S., Wright J.T. (2003). Seventh report of the Joint National Committee on Prevention, Detection, Evaluation, and Treatment of High Blood Pressure. Hypertension.

[8] Lerner M.G., Arbor A. (2006). Your Guide to Lowering Your Blood Pressure with DASH. DASH Eating Plan.

[9] Kwan M.W.-M., Wong M.C.-S., Wang H.H.-X., Liu K.Q.-L., Lee C.L.-S., Yan B.P.-Y., Yu C.-M., Griffiths S.M. (2013). Compliance with the Dietary Approaches to Stop Hypertension (DASH) diet: A systematic review. PLoS ONE.

[10] Blumenthal J.A., Epstein D.E., Sherwood A., Smith P.J., Craighead L., Caccia C., Lin P.H., Babyak M.A., Johnson J.J., Hinderliter A. (2012). Determinants and Consequences of Adherence to the Dietary Approaches to Stop Hypertension Diet in African-American and White Adults with High Blood Pressure: Results from the ENCORE Trial. J. Acad. Nutr. Diet..

[11] Fung T.T., Chiuve S.E., McCullough M.L., Rexrode K.M., Logroscino G., Hu F.B. (2008). Adherence to a DASH-style diet and risk of coronary heart disease and stroke in women. Arch. Intern. Med..

[12] Park Y.M.M., Steck S.E., Fung T.T., Zhang J., Hazlett L.J., Han K., Lee S.H., Kwon H.S., Merchant A.T. (2017). Mediterranean diet, Dietary Approaches to Stop Hypertension (DASH) style diet, and metabolic health in U.S. adults. Clin. Nutr..

[13] Kim H., Rebholz C.M., Garcia-Larsen V., Steffen L.M., Coresh J., Caulfield L.E. (2020). Operational Differences in Plant-Based Diet Indices Affect the Ability to Detect Associations with Incident Hypertension in Middle-Aged US Adults. J. Nutr..

[14] Schwingshackl L., Chaimani A., Schwedhelm C., Toledo E., Pünsch M., Hoffmann G., Boeing H. (2018). Comparative effects of different dietary approaches on blood pressure in hypertensive and pre-hypertensive patients: A systematic review and network meta-analysis. Crit. Rev. Food Sci. Nutr..

[15] Willett W.C. (2013). Nutritional Epidemiology: 24-Hour Dietary Recall and Diet Records Methods.

[16] Willett W. (2013). Nutritional Epidemiology: Chapter Food Frequency Questionnaire pg 70-92.

[17] Fuchs F.D., Fuchs S.C., Moreira L.B., Gus M., Nóbrega A.C., Poli-de-Figueiredo C.E., Mion D., Bortoloto L., Consolim-Colombo F., Nobre F. (2011). Prevention of hypertension in patients with pre-hypertension: Protocol for the PREVER-prevention trial. Trials.

[18] Fuchs F.D., Fuchs S.C., Moreira L.B., Gus M., Nóbrega A.C., Poli-de-Figueiredo C.E., Mion D., Bortolotto L., Consolim-Colombo F., Nobre F. (2011). A comparison between diuretics and angiotensin-receptor blocker agents in patients with stage I hypertension (PREVER-treatment trial): Study protocol for a randomized double-blind controlled trial. Trials.

[19] Cade J., Thompson R., Burley V., Warm D. (2002). Development, validation and utilisation of food-frequency questionnaires —A review. Public Health Nutr..

[20] Conway J.M., Ingwersen L.A., Moshfegh A.J. (2004). Accuracy of dietary recall using the USDA five-step multiple-pass method in men: An observational validation study. J. Am. Diet. Assoc..

[21] Godwin S.L., Chambers E., Cleveland L. (2004). Accuracy of reporting dietary intake using various portion-size aids in-person and via telephone. J. Am. Diet. Assoc..

[22] Monteiro J.P. (2007). Consumo Alimentar. Visualizando Porções.

[23] Pinheiro A.B.V., Lacerda E.M.A., Benzecry E.H., Gomes M.C.S. (2005). Tabela Para Avaliação de Consumo Alimentar em Medidas Caseiras.

[24] Fuchs S.C., Moreira L.B., Camey S.A., Moreira M.B., Fuchs F.D. (2008). Clustering of risk factors for cardiovascular disease among women in Southern Brazil: A population-based study. Cad. Saude Publica.

[25] Henn R.L., Fuchs S.C., Moreira L.B., Fuchs F.D. (2010). Development and validation of a food frequency questionnaire (FFQ-Porto Alegre) for adolescent, adult and elderly populations from Southern Brazil. Cad. Saude Publica.

[26] Rossato S.L., Fuchs S.C. (2021). Dietary data collected using 48-hour dietary recall: Within- and between-person variation. Front. Nutr..

[27] Rossato S.L., Olinto M.T.A., Henn R.L., dos Anjos L.A., Bressan A.W., Wahrlich V. (2010). Seasonal effect on nutrient intake in adults living in Southern Brazil. Cad. Saude Publica.

[28] Ministério da Saúde (2015). Alimentos Regionais Brasileiros.

[29] De Souza A.M., Pereira R.A., Yokoo E.M., Levy R.B., Sichieri R. (2013). Most consumed foods in Brazil: National Dietary Survey 2008–2009. Rev. Saude Publica.

[30] Blanton C.A., Moshfegh A.J., Baer D.J., Kretsch M.J. (2006). The USDA automated multiple-pass method accurately estimates group total energy and nutrient intake. J. Nutr..

[31] Rossato S.L., Fung T.T., Rodrigues M.P. (2017). A Data Entry System for Dietary Surveys Based on Visual Basic for Applications Programming. J. Acad. Nutr. Diet..

[32] Cole T.J., Bellizzi M.C., Flegal K.M., Dietz W.H. (2000). Establishing a standard definition for child overweight and obesity worldwide: International survey. Br. Med. J..

[33] Cole T.J., Flegal K.M., Nicholls D., Jackson A.A. (2007). Body mass index cut offs to define thinness in children and adolescents: International survey. Br. Med. J..

[34] Bland J.M., Altman D.G. (1999). Measuring agreement in method comparison studies. Stat. Methods Med. Res..

[35] Teufel N.I. (1997). Development of culturally competent food-frequency questionnaires. Am. Soc. Nutr..

[36] Samet J.M., Humble C.G., Skipper B.E. (1984). Alternatives in the collection and analysis of food frequency interview data. Am. J. Epidemiol..

[37] Apovian C.M., Murphy M.C., Cullum-Dugan D., Lin P.H., Gilbert K.M., Coffman G., Jenkins M., Bakun P., Tucker K.L., Moore T.J. (2010). Validation of a web-based dietary questionnaire designed for the DASH (Dietary Approaches to Stop Hypertension) diet: The DASH Online Questionnaire. Public Health Nutr..

[38] Niedzwiedzka E., Wadolowska L., Kowalkowska J. (2019). Reproducibility of A Non-Quantitative Food Frequency Questionnaire (62-Item FFQ-6) and PCA-Driven Dietary Pattern Identification in 13-21-Year-Old Females. Nutrients.

[39] Martela K., Kuźniewicz R., Pluskiewicz W., Tabor E., Zagórski P. (2019). Relevance of the semi-quantitative short Food Frequency Questionnaire in assessment of calcium consumption by female inhabitants of Zabrze over the age of 55 years (the Silesia Osteo Active Study). Arch. Osteoporos..

[40] Bredin C., Naimimohasses S., Norris S., Wright C., Hancock N., Hart K., Moore J.B. (2020). Development and relative validation of a short food frequency questionnaire for assessing dietary intakes of non-alcoholic fatty liver disease patients. Eur. J. Nutr..

[41] Affret A., Wagner S., El Fatouhi D., Dow C., Correia E., Niravong M., Clavel-Chapelon F., De Chefdebien J., Fouque D., Stengel B. (2017). Validity and reproducibility of a short food frequency questionnaire among patients with chronic kidney disease. BMC Nephrol..

[42] Akoglu H. (2018). User’s guide to correlation coefficients. Turkish J. Emerg. Med..

[43] Imaeda N., Goto C., Sasakabe T., Mikami H., Oze I., Hosono A., Naito M., Miyagawa N., Ozaki E., Ikezaki H. (2021). Reproducibility and validity of food group intake in a short food frequency questionnaire for the middle-aged Japanese population. Environ. Health Prev. Med..

